# Integrating aeration and rotation processes to accelerate composting of agricultural residues

**DOI:** 10.1371/journal.pone.0220343

**Published:** 2019-07-25

**Authors:** Fahad N. Alkoaik

**Affiliations:** Department of Agricultural Engineering, College of Food and Agriculture Sciences, King Saud University, Riyadh, Saudi Arabia; Gifu University, JAPAN

## Abstract

The active phase of conventional static composting systems varies dramatically, ranging from several weeks to several months. Therefore, this study was to examine the effect of a combined continuous aeration-rotation process on shortening the active phase of composted material. A mixture of tomato plant residues with 20%-chicken manure (*v*/*v*) was composted in two identical pilot-scale bioreactors. One of them was static, and the other was continuously rotated at 3 rpm; each was supplied with continuous aeration. Compost temperatures (*T*_c_) were measured throughout the composting process; the moisture content (*MC*) and carbon/nitrogen ratio (*C/N*) were measured at the beginning and end of the experiment. The quality and stage of compost were evaluated at the end of the experiment using Dewar, Solvita, and visual tests. Continuous aeration-rotation significantly reduced the active phase period to 4.5 days, increased the compost temperature (*T*_c_) to 60°C after 3 days of operation, and remained at 50–65°C for approximately 3 consecutive days (thermophilic stage). In contrast, compost in the static bioreactor remained in the mesophilic stage (*T*_c_ < 45°C). During the composting process, the *C/N* ratio was reduced from 30/1 to 23/1 in the rotating bioreactor, while it remained at 30/1 in the static bioreactor, indicating that the nitrogen content was not a limiting factor affecting the composting process. The *MC* was within the optimum range for microorganisms (58–61%) for both bioreactors. After the active phase had ended in the rotating bioreactor, the compost was inactive and ready for further maturation, while compost from the static bioreactor was still immature and active. These results show that the proposed method can be done on a commercial scale to significantly reduce the composting period and to enhance the compost stability and productivity.

## Introduction

Tomato (*Solanum lycopersicum* Mill.) production represents approximately 50% of the total greenhouse vegetable production (8000 hectares) in Saudi Arabia [1]. Greenhouse tomatoes produce approximately 15 tons of fresh plant residues per hectare per year, which makes plant residues among the most plentiful biomasses suitable for use as a compost material [2]. The large amount of plant residues that are produced by pruning and post-crop harvesting can be considered a sustainable source of organic matter to be composted and returned back to the soil as horticultural growth media [3, 4, 5]. Adding compost to soil improves soil structure by increasing organic matter, thereby improving soil fertility [6, 7].

A common composting method is open field composting, in which residues are left in windrows or static piles. However, these methods cannot be used in the Arabian Peninsula due to the high daily water evaporation rate (15 mm day^-1^ in July with a yearly increasing trend) and water shortage [8]. A more efficient and promising technique is composting in enclosed (in-vessel) systems using bioreactors. Bioreactors can process large amounts of waste in a limited space and can accommodate any type of organic waste (e.g., meat, animal manure, bio-solids and food scraps). This allows good control of the environmental factors, i.e., temperature, moisture content and airflow rate [9]. Moreover, bioreactor systems produce compost in a relatively short time, and are more efficient at breaking down materials, decreasing unpleasant odors and preventing disease transmission [10]. It is well known that aeration and rotation are the most critical operational parameters affecting the aerobic composting process. Mechanically forced aeriation is often used in both open and enclosed composting systems. Because excessive or insufficient aeration can adversely affect decomposition [11]; therefore, it is necessary to adjust aeration to the appropriate level for successful composting. In windrows and static piles, oxygen was found to be consumed within two hours after a pile was manually or mechanically turned; making the reaction anaerobic and inefficient [12, 13, 14, 15]. On the other hand, rotating bioreactors provide good mixing and uniform temperature distribution and produce a quick, consistent and uniform end product without any odor or leachate-related problems [11, 16, 17, 18, 19, 20, 21]. The combined rotation-aeration techniques may provide a favorable environment with a sufficient amount of oxygen and bioavailability of organic material that allows aerobic microbes to decompose the waste quickly. However, few studies have examined intermittent manual rotation with natural aeration for enclosed bioreactors. For example, a pilot-scale rotary drum bioreactor was manually turned three revolutions at fixed (6-, 12-, 18-, and 24-hr) intervals [16]. It was found that turning at 24-hr intervals provided the best composting performance. Other studies experimented with various types of materials using different numbers of rotations at 24-hr intervals, including one [17], two [19], three [20], four [18] and six [21] rotations. The previous studies maintained aerobic conditions by opening the side doors on the upper half of the drums for a period of time after the turning process. Successful composting requires sufficient oxygen distribution (via aeration-rotation) and adjustment of control parameters such as composting temperature (*T*_c_), moisture content (*MC*) and the *C/N* ratio [7]. In addition, specific biological and chemical tests are required to evaluate the compost quality and quantity [5, 22].

Little is known about the impact of continuous aeration-rotation on bioreactor performance, the composting process and the duration of the active phase. However, a continuous aerating-rotating pilot-scale bioreactor was recently used to evaluate the heat generation and losses during the composting process [23]. The active phase duration can be defined as “the period that starts at the end of the lag period and ends when the combined aeration-rotation no longer reheats a compost having *C/N* ratio below 25/1 and *MC* between 40% and 60%”.

Accordingly, the objective of this study was to evaluate the ability of a combination of continuous aeration and rotation to accelerate composting by reducing the active phase period for composting plants residues in a rotary bioreactor. To this end, we used two identical pilot-scale bioreactors, one static and one rotating at 3 rpm. We evaluated (i) the uniformity of compost temperature, (ii) changes in compost temperature with time and the length of the active phase, and (iii) *MC*, *C/N* ratio and compost stability and maturity indexes. We also developed a visual test that farmers could use to evaluate compost quality.

## Materials and methods

### Compost materials

Residues of greenhouse tomato plants (leaves, stems and some green and damaged fruits) were collected from various projects in the Riyadh area of Saudi Arabia. Prior to the composting process, the collected tomato residues had average moisture content (*MC*) of approximately 90%. Then, they were spread out on the ground to dry for three days, where their *MC* was reduced to 60%. To promote better aeration and *MC* distribution, the residues were chopped using a shredder (model FYS-76 Shredder, Mainland, Zhejiang, China). Furthermore, grinding was performed to decrease the particle size to approximately 1–2 cm to promote microbial degradation. The ground residues were left on the floor to dry out for two more consecutive days (*MC* was reduced to 15%), then tightly bagged and transported to the educational farm of the Agricultural Engineering Department, King Saud University, Riyadh, Saudi Arabia (Waste Management Lab). Thermally treated chicken manure (20% *MC*, 2% *N*) was obtained from a broiler project in the Riyadh area. Before starting the experiment, a mixture of tomato plant residues with 20% chicken manure was prepared. The mixing ratio was determined according to the recommended optimum *C/N* ratio of 30/1, and the *MC* of the final mixture was also adjusted to 60% as recommended [24]. The two bioreactors were each loaded with 50 kg of the final mixture.

### Description of the bioreactors

**Fig 1:**
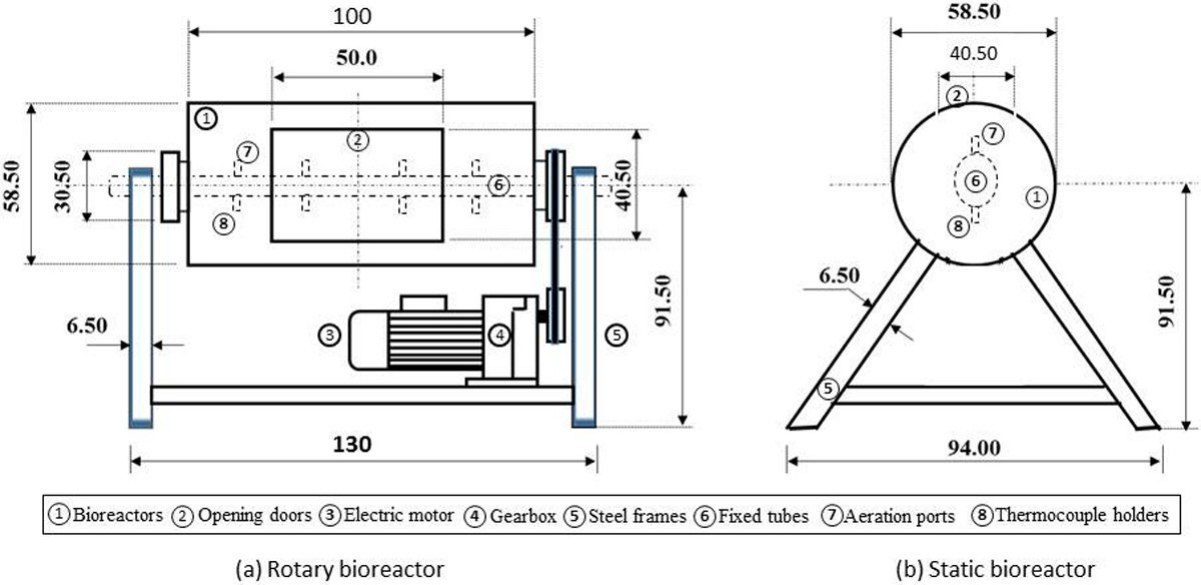
Schematic diagram showing the constructed (a) rotating bioreactor and (b) static bioreactor systems; dimensions in cm, not to scale.

Two identical pilot-scale bioreactors, each with a volume of 0.2 m^3^, were constructed at the educational farm of the Agricultural Engineering Department, King Saud University. Each bioreactor was a steel barrel with an inner diameter of 585.0 mm, a length of 914.4 mm and a wall thickness of 3.0 mm. Each bioreactor was designed to provide a space for 50 kg (wet weight) of compost mixture, leaving 25% of the volume as a headspace. In each bioreactor, a steel tube with a 50 mm outer diameter was installed horizontally at the centerline of the barrel for aeration and temperature measurement. In each bioreactor, a 50.0 × 40.5 cm door was constructed for loading, unloading, sampling and cleaning. A rubber gasket lining was fixed on the inner side of each door to prevent leakage. The outer surfaces of each bioreactor were insulated with a 25 mm thick glass wool blanket layer. For comparison, one bioreactor was static and the other was rotated horizontally around a fixed axis (i.e., a steel tube with a 50 mm outer diameter) at 3.0 rpm using a 0.25 hp electric motor (Model No. 220-380-3, Zhejiang, China). For aeration purposes, the perimeter of the tube included holes distributed longitudinally along the upper surface of the tube in the rotating bioreactor and in the lower surface in the static bioreactor. Layout dimensions for the rotary and fixed bioreactors, installed on steel frames with a rotating motor (for the rotary one), are illustrated (not to scale) in Fig 1A and 1B.

### Experiments and measurements

**Fig 2:**
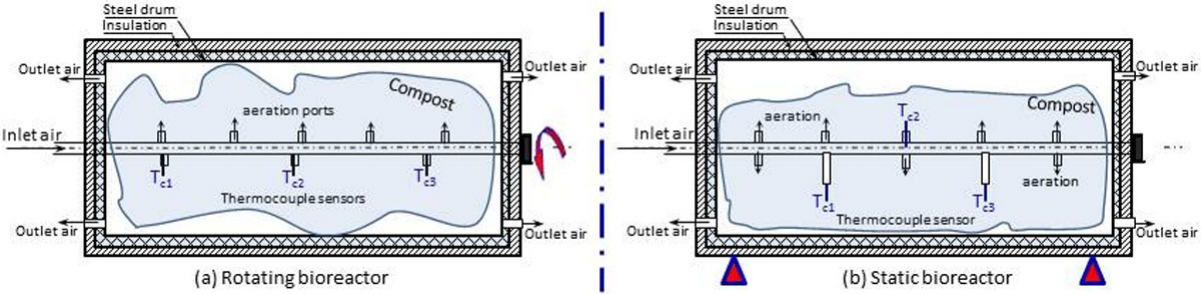
Cross-sectional views of the (a) rotating bioreactor and (b) static bioreactor Showing the inlet and outlet aeration ports and the locations of thermocouple sensors.

Compressed air was continuously supplied to each bioreactor at a flow rate of 0.005 m^3^ min^−1^ from a reservoir (10 bar, 0.2 m^3^ volume) connected to an air compressor (Model: Airmac, CRM203, 2.2 kW, Parkinson, Australia). The compressed air was supplied to the horizontal tubes around which the bioreactor rotated. The compressed air passed through flow meters (one for each bioreactor) to adjust the proper air flow rate and then to the compost via holes that were made in the horizontal tube (Fig 2A and 2B). The temperature of the compost (*T*_c_) was measured using three copper-constantan thermocouples (type-T, Cole Parmer, Chicago, IL, USA) fixed longitudinally at three locations above and below the horizontal tube. In the tube, aeration holes were on the opposite side of the thermocouple sensors to be far enough from the inlet air to reduce the negative impact of the air on the temperature measurements. Aeration ports were located downward in the rotating bioreactor (Fig 2A) and downward and upward in the static bioreactor (Fig 2B). The thermocouple wires were placed inside the tube to the outside and connected to a portable data logger (Model: Testo 177-T4 V01-02). Ambient temperature (*T*_am_) was measured with a Thermo-Hygrometer DMA033 (LSI-Lastem, Milano, Italy). The measured parameters were recorded every 10 seconds, averaged for every 10 min period and saved in the data logger.

### Calibration test

**Fig 3:**
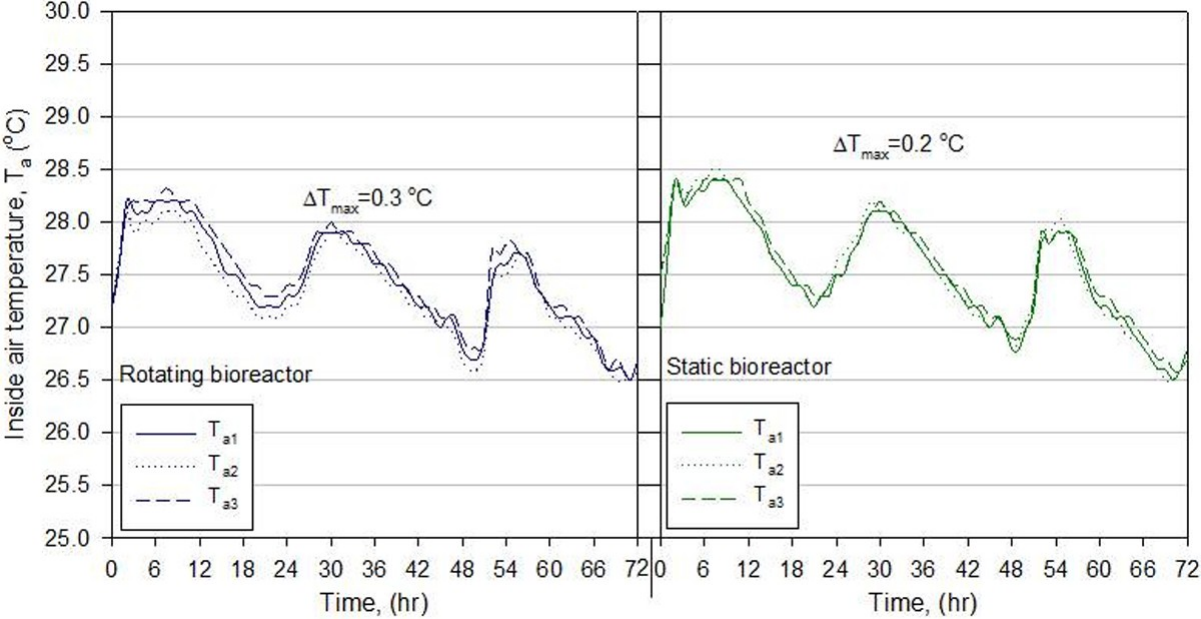
Time course of air temperatures recorded by the three sensors (*T*_a1_, *T*_a2_, and *T*_a3_) fixed at three different locations inside the rotating and static bioreactors.

To ensure that the temperature sensors were working properly and for accurate comparisons between the rotating and static bioreactors, before starting the experiment, the two bioreactors were operated empty and the air temperatures were measured by the three sensors in each bioreactor (*T*_a1_, *T*_a2_, and *T*_a3_) and recorded over 72 hours and illustrated in Fig 3. During 72 hours, the maximum temperature difference among the three sensors ΔT_max_ was 0.2°C (in the static bioreactor) and 0.3°C (in the rotating bioreactor). The temperature levels in the rotating bioreactor were somewhat lower than those in the static bioreactor because the rotation and air mixing in the rotating bioreactor may have increased the heat loss to the air outside the bioreactor. Based on the average air temperature [(*T*_a1_+*T*_a2_+*T*_a3_)/3] in each bioreactor, the maximum temperature difference during 72 hours was estimated to be 0.33°C, which is considered the maximum expected error in the comparison of temperature.

### Determining the compost parameters

Moisture content (*MC*, %) was measured by the oven-drying method (ASTM procedure D3173-73). A representative compost sample was placed in an air oven at 105°C for 24 hours, until a constant weight was achieved. The total organic carbon (*TOC*) content was calculated assuming that it was equal to 55% of the organic matter (*OM*), according to Haug [24] as:

OM(%)=100−Ash(%)andTOC(%)=OM(%)×0.55 (1)

Total nitrogen (*TN*, %) was analyzed based on the Kjeldahl method using Foss-Kjeltec (Model: 8100, Denmark). Consequently, the *C/N* ratio was calculated using the values of *TOC* and *TN*. The moisture content (*MC*, %) and carbon to nitrogen (*C/N*) ratio were determined at the beginning and end of the experiment (day 0 and day 8). To determine the maturity level of the composted material, three samples were taken randomly from each bioreactor just after taking the compost material out of the bioreactors (at day 8) for testing and evaluation.

For the Solvita test, the moisture content (*MC*) of the tested samples (100 mL of composted materials) was adjusted to 60%, and the samples were incubated in 200 mL containers with a Solvita reactor for 4 hours following the manufacturer’s instructions [25]. Then, the extent of color change was measured using the new DCR (Digital CO_2_ & NH_3_ Color Reader, Solvita).

For the Dewar self-heating test [26], the six samples were stored at 4°C until use. Each sample was kept in a flask for six days, then the maximum temperature (*T*_max_) and the corresponding room temperature (*T*_room_) were recorded, and consequently, the temperature difference ΔT (ΔT = *T*_max_−*T*_room_) was estimated.

For visual testing, the color components of each compost sample were determined on days 0 and 8 using a ColorFlex spectrophotometer (Hunter Lab-ColorFlex, Hunter Associates Laboratory, Inc.-Reston, US). Each sample was placed in a sample cup, and then the cup was covered and inserted into the measuring chamber. Consequently, color components were measured where L* is the lightness or darkness (black: L* = 0; white: L* = 100), +a* is redness, -a* is greenness, +b* is yellowness and–b* is blueness. The average value of each component was obtained for the three samples from each bioreactor. The measuring device was calibrated with standard calibration plates provided by the manufacturer. The compost color change (ΔE) during the composting process (8 days) was determined for each bioreactor using the color difference equation [27]:

$$\Delta E=\sqrt{{\left(L_{o}^{*}-L^{*}\right)}^{2}-{\left(a_{o}^{*}-a^{*}\right)}^{2}+{\left(b_{o}^{*}-b^{*}\right)}^{2}}$$ (2)

where $L_{o}^{*},\;a_{o}^{*}\;b_{o}^{*}$ are the color components measured at the initial stage (day-0) and L*, a*, b* are the color components measured at the final stage (day-8).

## Results and discussion

The main parameters affecting the composting process of agricultural residues are oxygen, moisture content (*MC*), carbon to nitrogen ratio (*C/N*) and compost temperature (*T*_c_). In the present study, we monitored *MC*, *C/N* ratio and *T*_c_ but not oxygen because both bioreactors had a continuous supply of oxygen that makes oxygen isn’t a limiting factor controlling the process.

### Moisture content (MC)

The optimum moisture content (*MC*) of compost is a vital factor for the microbial decomposition of organic waste. The initial *MC* of approximately 60% is proper for an acceptable composting [28]. However, excessive moisture content leads to a lower rate of oxygen resulting in anaerobic conditions that decrease the organic matter degradation rate. In the present study, however, the *MC* of the composted material was estimated on the 1^st^ and 8^th^ days for each bioreactor. During the experiment, *MC* remained in the range 57.8% - 60.0% in the rotating bioreactor and in the range 60.0% - 61.4% in the static bioreactor, which was in the optimum range for microbial activity [29]. Therefore, it can be concluded that the *MC* was not a limiting factor during the composting process in either bioreactor.

### Carbon to nitrogen ratio (*C/N*)

The *C/N* ratio is used to describe organic waste decomposition and compost quality with respect to organic matter and *N* cycling. Several studies have indicated that for high-quality final compost, the *C/N* ratio should be in the range from 12/1 to 25/1, for example [30, 31]. In compost with a high *C/N* ratio (> 40/1), microorganisms take considerable time to break down waste because a deficiency of *N* reduces composting performance [32]. However, compost with a low *C/N* ratio causes ammonium toxicity [33]. In the present study, the *C/N* ratio decreased from 30/1 to 23/1 (in the rotating bioreactor) and remained at 30/1 (in the static bioreactor) during the composting process. Bazrafshan et al. (2016) [33] attribute the reduction in the *C/N* ratio during the composting process to the transformation of carbon to CO_2_ followed by a reduction in the organic acid concentration and an increase in the *N* content per unit material. Therefore, *N* was not a limiting factor during the composting process in either bioreactor.

### The compost temperature (*T*_c_)

**Fig 4:**
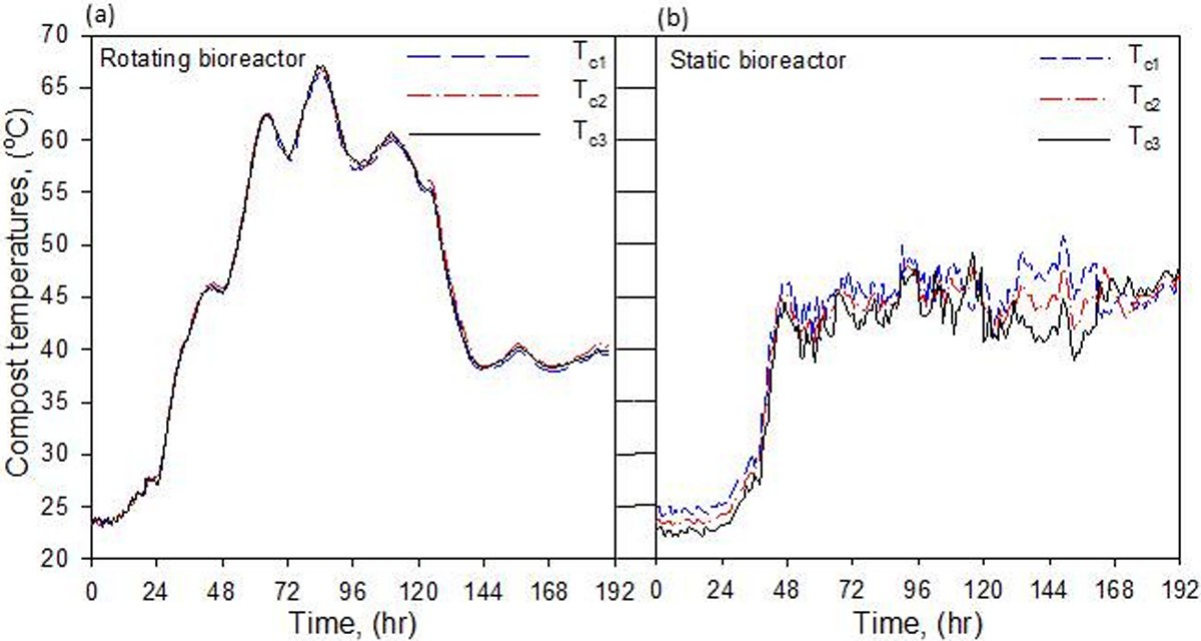
Time course of compost temperatures recorded by the three sensors (*T*_c1_, *T*_c2_, and *T*_c3_) fixed at three different locations inside the rotating and static bioreactors.

Composting is an exothermic process that generates heat due to the aerobic metabolic reactions of the composting materials. This, in turn, increases the temperature of the compost and the bioreactor components. Mixing of compost materials in the rotating bioreactor created uniform temperature distribution of compost. Therefore, no significant differences were observed among the three temperatures of compost (*T*_c1_, *T*_c2_ and *T*_c3_) measured in rotating bioreactor (Fig 4A). While, in the static bioreactor, a maximum temperature difference of 5°C was observed among the three compost temperatures (Fig 4B). In the upcoming discussion, the average of the three compost temperatures was used to represent the compost temperature (*T*_c_) in each bioreactor.

**Fig 5:**
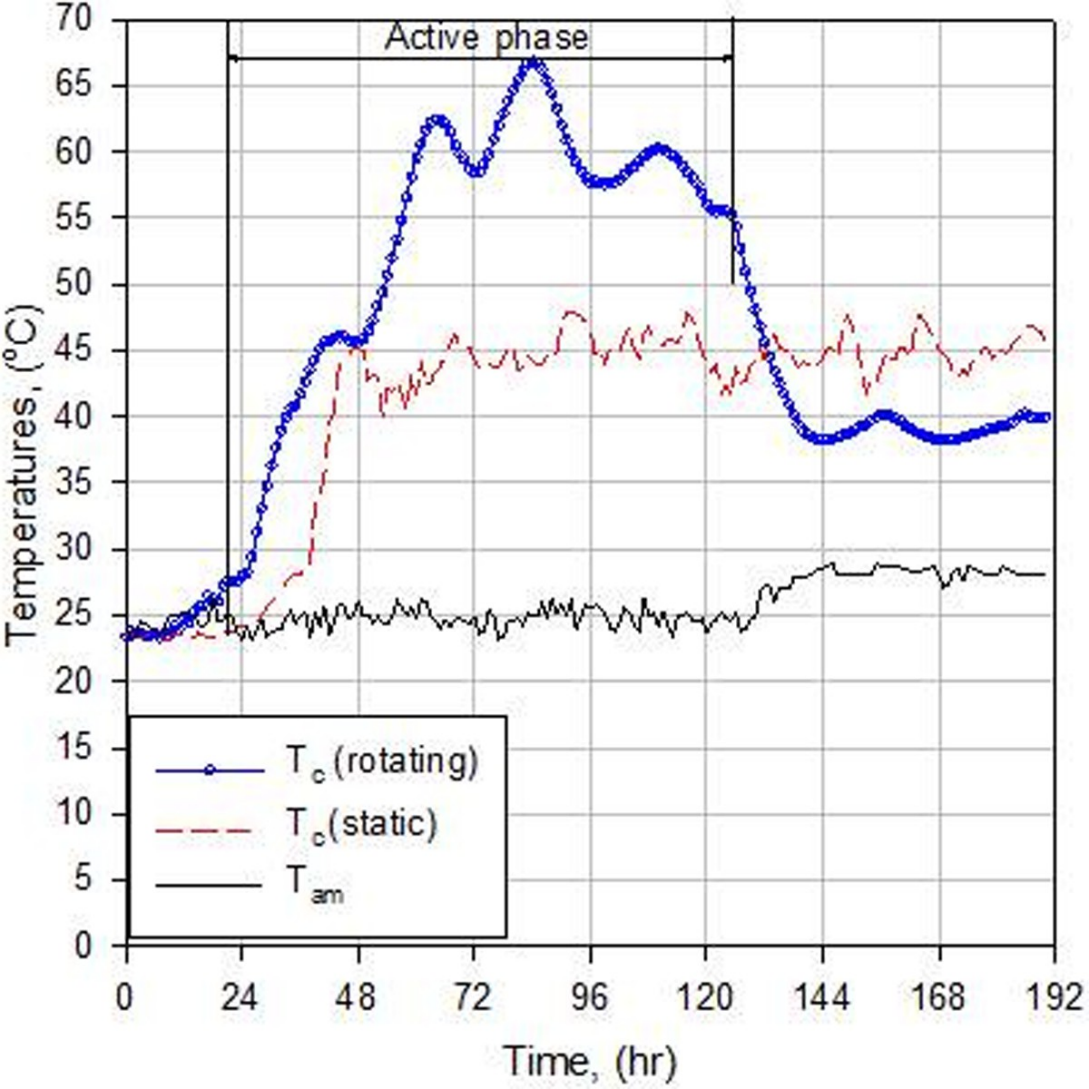
Time course of compost temperatures measured in the rotating and static bioreactors (*T*_c_) and for the ambient air (*T*_am_) during the composting process.

The time course of the average compost temperatures (*T*_c_) and ambient air temperature, (*T*_am_) in the static and rotating bioreactors are illustrated in Fig 5. Rotation provides optimal conditions for the composting process to proceed through the three standard phases, enhanced the metabolic exothermic reactions, and increased the internal energy of the compost. Thus, rotation created optimal composting conditions and increased the breakdown of the available organic matter and nitrogenous compounds through microbial activity. This rapidly increased *T*_c_ to approximately 55°C after 3 days, where it remained for approximately three days in the thermophilic stage (Fig 5). In the static bioreactor, microbial activity was depressed, lowering *T*_c_ to the mesophilic stage during the entire experiment (Fig 5). Moreover, *T*_c_ was generally below 50°C, which increases the risk of weed seed survival and plant pathogens in the end product. Mixing the compost materials in the rotary bioreactor significantly reduced the active phase period (high-temperature phase) to less than 3 days compared to several weeks, or possibly several months, for the static composting systems. In addition, keeping *T*_c_ in the range of 50–65°C for 2–3 consecutive days is sufficient to meet the optimum requirements for the destruction of pathogens and weed seed viability [24]. Temperatures above 65°C may inactivate most of the beneficial decomposing microorganisms (fungi, actinomycete and some bacteria), limiting further decomposition to thermophilic spore forming bacteria [34]. However, in the rotating bioreactor, high temperature (> 65°C) occurred for only a few hours and quickly declined (Fig 5), which was not enough to kill the microorganisms. On the fifth day, *T*_c_ decreased to below 55°C indicating that bioavailable carbon was starting to be depleted.

Based on the previous discussion, Oxygen, *MC* and *C/N* ratio were not limiting factors affecting the composting process; however, the compost temperature can be used as an indirect measured of microbial activity [34]. Therefore, in the rotating bioreactor, the level of microbial heat generation due to composted material degradation was clearly reduced after the bioavailable carbon was utilized. Hence, microbial activity declined, resulting in a decreased compost temperature below 40°C at the end of the active phase of the composting process.

### Thermal kinetics of compost

During the composting process, the heat was generated as a result of the degradation of organic matter, first raising the compost temperature (*T*_c_) to the mesophilic stage (25–45°C) and then to the thermophilic stage (45–67°C), as shown in Fig 5. The maximum temperature in the rotating bioreactor was 66.8°C and was achieved after 84 hours. Temperature above 55°C (which is required for destruction of pathogens and weed seeds) were maintained for 68 hours. However, in the static bioreactor, the maximum temperature was 52°C, which was achieved within a short period after 114 hours, and the compost temperature during most of the composting process did not exceed that of the mesophilic stage.

**Fig 6:**
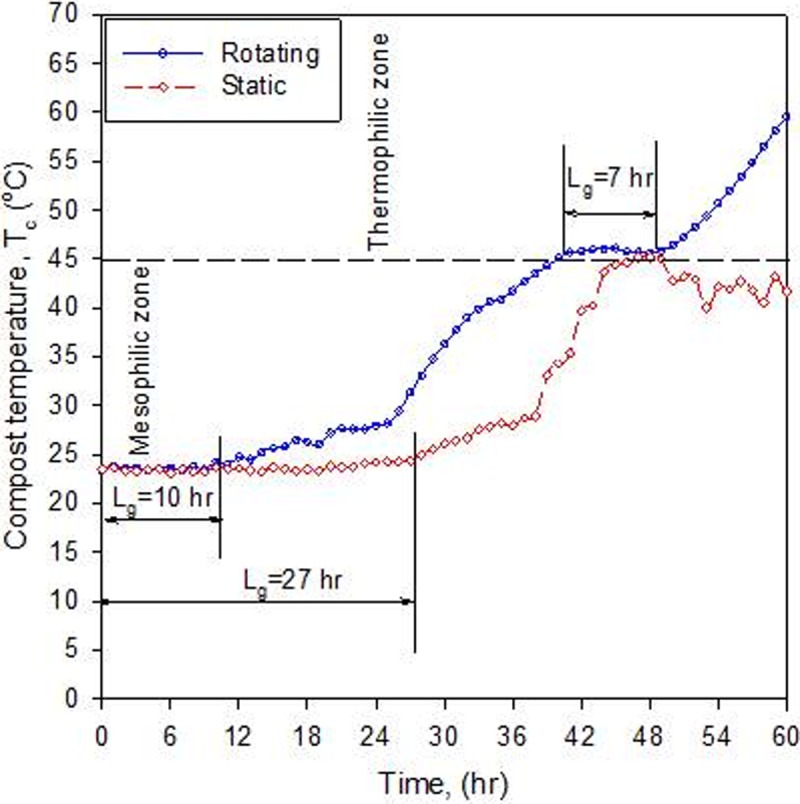
Increase of compost temperature and the mesophilic and thermophilic lag periods estimated for the rotating and static bioreactors.

Fig 5 shows three distinct phases; thee lag, active (mesophilic and thermophilic), and maturation (curing) phases. The initial mesophilic lag period (microbial adaptation) was reduced to 10 hours in the rotating bioreactor compared with 27 hours in the static (Fig 6). The thermophilic lag period (second lag) was 7 hours in the rotating bioreactor, whereas there was no second lag period in the static bioreactor because the compost temperature did not exceed 45°C (Fig 6). This indicated that rotating the compost decreased the mesophilic lag period by at least 37%. The transition from lag phase to active phase is determined by an exponential increase in temperature (an indirect measurement of microbial activity). In general, compost temperature increased more rapidly in the rotating bioreactor than in the static bioreactor. Table 1 compares thermal kinetic parameters in the two bioreactors.

**Table 1:** Thermal kinetic parameters estimated for the rotating and static bioreactors.

Parameter (unit)	Rotating bioreactor	Static bioreactor
**Lag period (hr)**		
Mesophilic	10	27
Thermophilic	7	-
**Rate of temperature increase (°C hr**^**-1**^**)**		
Mesophilic	0.33 for 15 hours 1.25 for 13 hours	0.42 for 12 hours 2.6 for 8 hours
Thermophilic	1.7	-
**Maximum temperature (°C)**	66.8	52
**Peak time (hr)**	84	114
**Duration of temperature (hr)**		
≥ 35°C	160	150
≥ 45°C	90	30
≥ 50°C	71	6
≥ 55°C	68	-
≥ 60°C	24	-

### Solvita maturity test

The results of Solvita test for the 6 composted samples taken from the two bioreactors on the 8^th^ day are illustrated in Table 2. According to Solvita maturity index [25], which ranges from 1 (unstable compost) to 8 (very stable and well-matured compost). Based on the values of CO_2_ and NH_3_, the index of the rotating bioreactor's compost was estimated to be 6. This means that the compost moved beyond the active phase of decomposition and was ready for curing, reducing the need for intensive handling. In contrast, the index was estimated to be 3 for the static bioreactor’s compost, which indicated that the compost was still active, fresh and still required intensive oversight and management.

**Table 2:** Results of the solvita test for six samples of compost taken from the static and rotating bioreactors.

Sample No.	Rotating bioreactor				Static bioreactor			
	C/N ratio	MC (%)	Solvita index		C/N ratio	MC (%)	Solvita index	
			CO_2_	NH_3_			CO_2_	NH_3_
1	23.2	58.1	5.07	4.3	29.4	62.3	4.54	3.43
2	22.8	57.4	5.12	4.35	31.2	61.2	4.43	3.47
3	23.3	57.8	5.15	4.29	29.7	60.9	4.51	3.4
Mean	23.1	57.7	5.11	4.31	30.1	61.4	4.49	3.43
SD	0.22	0.35	0.04	0.03	0.96	0.73	0.05	0.03

### Dewar test

Table 3 shows the results of the Dewar self-heating test results for the six samples taken from the two bioreactors on the 8^th^ day. The maximum temperature of compost samples taken from the rotating bioreactor was very close to room temperature. An average value of ΔT of 3.4°C, which is much lower than 10°C, indicated that the mixture completed the active phase and was ready for maturation [26]. In contrast, the compost samples taken from the static bioreactor showed higher increments of ΔT exceeding 10°C and reaching 17.3°C, on average. This indicated that the materials were still decomposing (active). These results demonstrate that rotating the compost can greatly reduce the time to achieve high-quality compost.

**Table 3:** Dewar self-heating test results for six compost samples taken from the static and rotating bioreactors.

Dewar self-heating test								
Sample No.	Rotating bioreactor				Static bioreactor			
	*T*_max_ (°C)	*T*_room_ (°C)	ΔT (°C)	Remarks	*T*_max_ (°C)	*T*_room_ (°C)	ΔT (°C)	Remarks
1	25.9	22.2	3.7	Compost class-A (ready for maturation stage)	38.8	21.3	17.5	Compost class-B (Mesophilic, still active, immature)
2	25.1	21.7	3.4		37.9	21.7	16.2	
3	24.4	21.2	3.2		39.1	20.9	18.2	
Mean	25.13	21.7	3.43		38.63	21.3	17.3	
SD	0.75	0.5	0.25		0.62	0.4	1.01	

### Visual test

**Fig 7:**
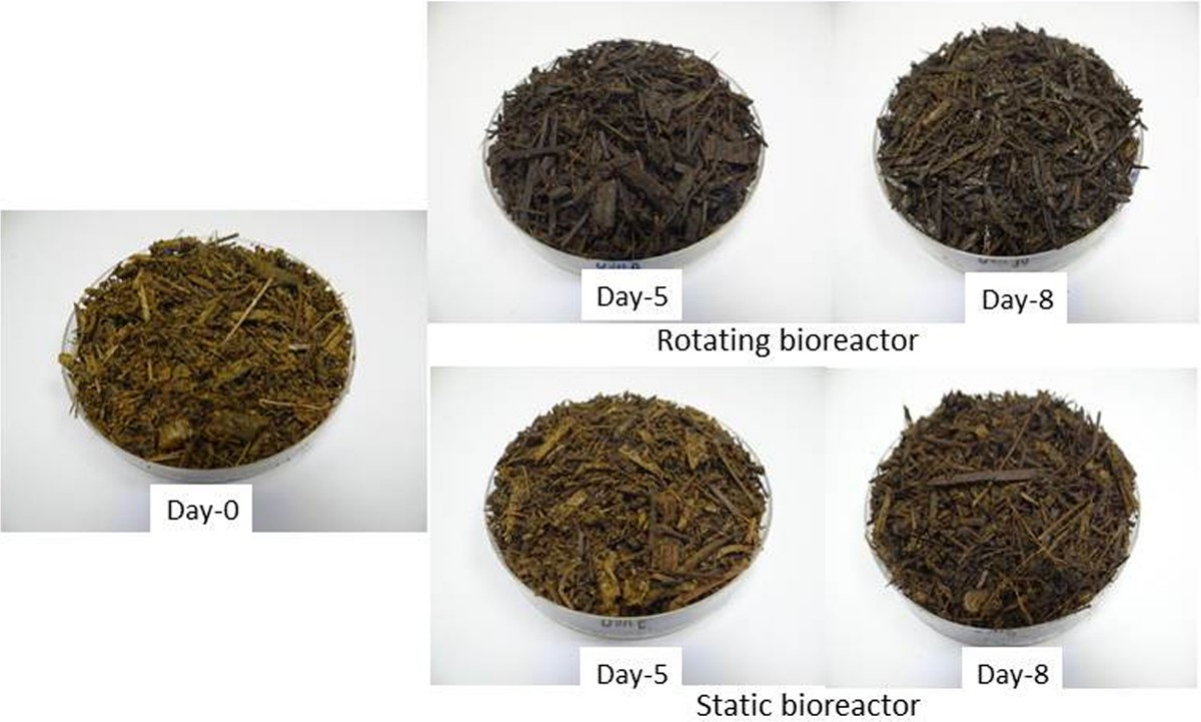
Photos of compost material taken from the two bioreactors at days 0, 5 and 8 to show the changes in the color and material structure (visual testing).

The texture of mature compost depends on the source materials and age. A practical and simple method that can be used by farmers to judge the maturity level of compost is the visual method, which involves observing the product with the human eye to assess its color, homogeneity and texture. Fig 6 illustrates photographs of the compost samples taken at days 0, 5 and 8 from the two bioreactors. Changes in the compost color are easily visible after 5 days of composting (Fig 7). Even though the tested samples were taken after the completion of the active phase period, the visual appearance and smell of the compost product were clearly different from those of the original sample (raw mixture). The texture of compost from the rotating bioreactor was finer, more crumby and has a strong smell of ammonia, while compost from the static bioreactor remained almost unchanged (Fig 7). The color components of the composted material can be used to describe the composting process and as an indicator of compost stability and maturity level [35]. Accordingly, to support the visual observation, as illustrated in Fig 7, the change of the color components of compost were measured at days 0 and 8; data of day 8 are summarized in Table 4 to represent the color differences. On day 8, the color index (L*) of the rotating bioreactor compost was lower than that of the static bioreactor compost (the darkness of the rotating bioreactor compost was increased). Moreover, the greenness (a*) and yellowness (b*) colors of compost were increased in the rotating bioreactor compost than those in the static bioreactor. In general, the total change in color (ΔE) was much higher in the rotating bioreactor than that in the static bioreactor. This simply indicated that rotating the bioreactor significantly improved the quality of compost and the maturity level compared with that of the static bioreactor compost.

**Table 4:** Results of the color test for six compost samples taken from the static and rotating bioreactors.

Compost color test at day-8										
Sample No.	Static bioreactor					Rotating bioreactor				
	L*	a*	b*	ΔE	Remarks	L*	a*	b*	ΔE	Remarks
1	22.6	4.6	7.7	8.5	Blackness, greenness and yellowness slightly increased. Texture and compost structure did not improve.	17.6	2.7	4	15.1	Blackness, greenness and yellowness increased. Texture and compost structure improved.
2	22.8	4.4	7.8	8.4		17.5	2.7	4	15.2	
3	22.7	4.5	7.5	8.4		17.5	2.7	4	15.2	
Mean	22.7	4.5	7.7	8.4		17.5	2.7	4	15.2	
SD	0.1	0.1	0.15	0.06		0.06	0.0	0.0	0.06	

### Practical implications of this study

One of the main challenges facing composting practices is the long duration of the active phase. This requires close attention and good management practice to oxygen requirements, turning frequency, temperature levels, and pathogens risk and odors control. Conventional composting systems require several weeks or even several months, in the static piles, to complete the active phase of composting and to be ready for further maturation. The obtained results from the present study showed that integrating the continuous rotation and aeration can significantly reduce the duration of the active phase to only 4.5 days, with a very short lag period (10h), and enough thermophilic stage to meet the guidelines for good compost that is free of plant pathogens. The improvement of the proposed composting protocol over other methods is illustrated in Table 5. According to the parameters reported in Table 5, the proposed method can be done on a commercial scale. Further studies are needed to examine different composting strategies for different agricultural residues. For example, composting might be improved by applying intermittent forced aeration-rotation (on-off) to the bioreactor, and applying natural aeration (via holes in the bioreactor body) combined with a continuous or intermittent rotation of the bioreactor.

**Table 5:** Results of the proposed method compared to results of other composting methods.

Composted material	Size (Liter)	Rotation protocol	Aeration protocol	Lag phase duration (hr)	Active phase duration^+^ (day)	*T*_c, max_ (°C)	Ref.
Cattle manure & green vegetables & sawdust	250	3 rotations/					
		6hr	Intermittent	Not reported	9	53	[16]
		12hr			11	53	
		18hr			9	55	
		24hr			8	58	
Different mixtures of vegetables wastes & cow manure & sawdust	550	1 rotation/24 hr	Intermittent	Not reported	8–12	61–66	[17]
Pig & poultry carcasses	360	24-min rotations/					
		1hr	Intermittent	Not reported	11	60	[21]
		2hr			16	62	
		3hr			17	70	
		4hr			18	70	
Penicillin mycelial dreg & sewage sludge & sawdust & rice straw	390	static	Continuous	Not reported	28	65	[14]
Tomato residues & chicken manure	200	Continuous	Continuous	10	4.5	66.8	Present study

+ The active periods were determined, based on the definition in this study, from the *T*_c_ evolution figures reported in the corresponding references.

## Conclusion

Continuous aeration-rotation of compost significantly reduced the active phase of composting to only 4.5 days; whereas several weeks or even several months may be required to finalize the active phase of the static compost. Unlike the static bioreactor, the rotating bioreactor with continues aeration increased and maintained the compost temperature uniformly distributed and in the range between 50–65°C for three consecutive days, achieving a successful active phase of the composting process. However, the compost in the static bioreactor remained in the mesophilic stage, and the compost temperature remained below 45°C during the experiment. Based on the Solvita, Dewar-self heating, visual and color tests, the compost in the rotating bioreactor completed the active phase and became inactive and ready for further maturation, while that of the static bioreactor was still active.

## Nomenclature

*a**: compost color index (+ ve is redness,—ve is greenness) (-)
*b**: compost color index (+ ve is yellowness,—ve is blueness) (-)
*C/N*: carbon to nitrogen ratio (-)
*d*: day
*L**: compost color index (0 black to 100 white) (-)
*L*: liter
hr: hour
*MC*: moisture content (%)
*NC*: nitrogen content (%)
*OM*: organic matter content (%)
*T*_a_: air temperature measured in the empty bioreactor (°C)
*T*_am_: ambient air temperature (°C)
*T*_c_: temperature of compost (°C)
*T*_c, max_: maximum compost temperature (°C)
*T*_max_: maximum temperature recorded by Dewar's flask (°C)
*T*_room_: room air temperature (°C)
*TN*: total nitrogen content (%)
*TOC*: total organic carbon (%)
ΔE: index of the color difference (-)
ΔT: temperature difference (*T*_max_-*T*_room_) (°C)
*v*: volume
